# The prevalence of gastro-esophageal reflux disease and esophageal dysmotility in Chinese patients with idiopathic pulmonary fibrosis

**DOI:** 10.1186/s12876-015-0253-y

**Published:** 2015-02-19

**Authors:** Feng Gao, Anthony Robert Hobson, Zhan Min Shang, Yan Xiang Pei, Yan Gao, Jian Xin Wang, Wan Nong Huang

**Affiliations:** 1 Digestive Department, Beijing Chao-Yang Hospital, Capital Medical University, No.8 Gong Ren Ti Yu Chang South Road, Chao Yang District Beijing, 100020 China; 2 The Functional Gut Clinic, London, W1G 6NB United Kingdom

**Keywords:** Gastro-esophageal reflux disease, Idiopathic pulmonary fibrosis, Esophageal function tests

## Abstract

**Background:**

The cause of idiopathic pulmonary fibrosis (IPF) remains unknown, yet gastro-esophageal reflux disease (GERD) is highly prevalent in this population. GERD prevalence was studied, and esophageal function tests (EFT) were assessed in Chinese IPF patients.

**Methods:**

We prospectively studied 69 IPF patients who undertook both stationary High Resolution esophageal Manometry/Impedance (HRiM) and 24-hour esophageal Multi-Channel Intraluminal Impedance with pH Recordings (MII/pH). Patients were divided into GERD+ and GERD- groups according to pH results. Controls were HRiM treated healthy volunteers, and patients without IPF received HRiM and MII/pH diagnosed with GERD.

**Results:**

69 IPF patients, 62 healthy volunteers, and 88 IPF negative GERD patients were selected. GERD prevalence in IPF was 43/69 (62.3%), and 58.1% of patients presented with at least one typical symptom. Symptoms had a sensitivity of 58.1%, a specificity of 61.6%, a positive predictive value of 71.4% and a negative predictive of 47.1%. Compared with healthy volunteers, IPF patients had significantly decreased lower esophageal sphincter pressure (LESP), upper esophageal sphincter pressure (UESP) and complete bolus transit rate (CBTR). By contrast, IPF patients had increased total bolus transit time and prevalence of weak peristalsis. MII/pH showed that one third of IPF patients had abnormal distal and proximal reflux, especially non-acid reflux. Compared with GERD patients without IPF, GERD patients with IPF had significantly decreased CBTR and UESP with increased bolus exposure time.

**Conclusions:**

GERD prevalence in IPF was high, but symptoms alone were an unreliable predictor of reflux. IPF patients had lower LESP and UESP, impaired esophageal peristalsis and bolus clearance function with more proximal reflux events.

## Background

Idiopathic pulmonary fibrosis (IPF) is a chronic and progressive fibrotic lung disease that is characterized by a histological pattern of interstitial pneumonia with a median survival from the time of diagnosis of 2 – 3 years [1-3]. Although the cause of IPF is unknown, gastro-esophageal reflux disease (GERD) is highly prevalent in this population [4-6], and emerging data supports a role for chronic micro-aspiration (e.g., subclinical aspiration of small droplets of refluxate), which may cause repetitive subclinical injury to the lung leading to pulmonary fibrosis [3,7].

The last ten years has been an exciting time in the study of esophageal motor disorders due in part to advances in esophageal function testing methodology. Techniques like manometry have enjoyed many improvements due to advances in transducer technology, computerized automation, advances in software development, and topographic color-plot data presentation [8-10]. In addition, the concomitant measurement of esophageal intraluminal impedance, which provides complementary data that details functional bolus transit during manometry without the need for radiation, has increased the clinical utility of esophageal function tests (EFT).

Using these techniques, we studied the prevalence of GERD and esophageal motility disorders in Chinese patients presenting with idiopathic pulmonary fibrosis (IPF) and compared these data to using symptomatic assessments alone.

## Methods

### Ethics

The study received ethics approval from the local Ethics Board of Beijing Chao-Yang Hospital, and the Capital Medical University, China. In addition, all participants gave written informed consent.

### Patient selection

This was a retrospective study that evaluated all patients who undertook stationary High Resolution esophageal Manometry/Impedance (HRiM), and 24-hour esophageal Multi-Channel Intraluminal Impedance with pH (MII/pH) recordings at the Digestive Department of Beijing Chao-Yang Hospital, Capital Medical University from July 2011 - July 2013. Chinese patients that presented with IPF were included in this study and treated at the respiratory Department of Beijing Chao-Yang Hospital, Capital Medical University. GERD patients without IPF were included as a control group. Patients with other chronic active medical diseases (e.g., patients that presented with coronary artery disease, hypertension, malignancy, diabetes mellitus, and connective tissue disease) were excluded. Normal HRiM values were taken from healthy volunteers at the Digestive Department of Beijing Chao-Yang Hospital, Capital Medical University from January 2010 - January 2011.

Each patient had an established diagnosis that was confirmed by a pulmonary medicine physician. The diagnosis of IPF met the American Thoracic Society (ATS) and European Respiratory Society (ERS) criteria for the diagnosis of IPF, which required the absence of an identifiable etiology for interstitial lung disease and a histopathological or radiological usual pattern of interstitial pneumonia [1,2].

### Data collection

Values of forced vital capacity (FVC) and diffusing capacity of the lung for carbon monoxide (DLCO) from pulmonary function tests were collected and analyzed. All patients answered a validated questionnaire with typical symptoms were defined as heartburn, regurgitation, chest pain and atypical symptoms such as cough, dyspnea on exertion, belch, difficulty swallowing, globus sensation, hoarseness and epigastric pain. Current use of acid reducing medications was also documented.

### Stationary high resolution esophageal manometry and impedance

A specially designed solid-state manometry catheter (Sandhill Scientific Inc., Highland Ranch, CO, USA) that was equipped with 32 manometric sensors and four pairs of MII sensors separated by a 5 cm interval, was used to determine esophageal pressures and impedance in patients that were examined in the supine position. The lower esophageal sphincter (LES) was examined with distal circumferential manometric sensors. The catheter was positioned so that the pressure transducers were located across the upper esophageal sphincter, esophageal body, and the LES; in addition the distal channels were positioned in the stomach. Ten oral administrations of 5 ml normal (0.9%) saline solution were then performed at 30-second intervals, followed by 10 additional swallows of a synthetic gel of known viscosity (EFT Viscous, Sandhill Scientific Inc., #500 Highland Ranch, CO, USA).

Measurements of esophageal physiology were standardized in recent years so that acquired data is comparable across sites and between research studies. The standardized protocol (adopting the Chicago Classification) requires that each patient orally ingests 10 × 5 ml liquid boli (at a rate of approximately one swallow per minute) in order to assess the parameters of normal peristalsis. Saline is used (as opposed to regular drinking water) as it increases the signal detected by electric impedance electrodes that track bolus transit. As liquid is relatively uncomplicated to clear for most people, an additional ‘peristaltic challenge’ is often used by swallowing a viscous gel, which better resembles normal food. This can often reveal abnormalities in peristalsis that is not seen with liquid.

Esophageal bolus clearance can be assessed by measurement of total bolus transit time (TBTT) by classifying swallows as a complete bolus transit (i.e., if the bolus entry occurs at the most proximal site, and bolus exit points are recorded in all three distal recording segments) or as incomplete bolus transit (i.e., if the bolus exit is not identified at any of the three distal recording segments), and complete bolus transit rate (CBTR) [11]. The distal contractile integral (DCI) of the distal segmental contraction is a parameter that integrates the length of the smooth muscle esophagus (cm), contractile pressure (mm Hg), and duration (s) of contraction. Distal esophageal amplitude (DEA) is an average of the contraction amplitude at 5 and 10 cm above the LES. Integrated relaxation pressure (IRP) reports mean EGJ pressure that is measured with an electronic equivalent of a sleeve sensor in four continuous or non-continuous seconds of relaxation in the ten-second window after deglutitive LES relaxation. The normal range for isobaric contour breaks was 0–20% for >5 cm breaks (“large break”) and 0–30% for 2–5 cm breaks (“small break”). Patients were classified as “normal peristalsis” if they had presented with a number of peristaltic breaks within the normal range or had no peristaltic breaks. Patients were classified with “weak peristalsis” if they had presented with 20-100% large breaks or 30-100% small breaks of peristalsis [8,12,13].

### 24-hour oesophageal multi-channel intraluminal impedance and pH recordings

The 2.1 mm outer diameter study catheter was comprised of six electrode pairs measuring the intraluminal impedance at 3, 5, 7, 9, 15, and 17 cm above the LES, and an antimony pH sensor that was 5 cm above the LES (Sandhill Scientific Inc., Highland Ranch, CO, USA). An impedance amplifier, delivered an ultra-low current over a range of 1–2 KHz with resulting current flow variations in response to changes in intraluminal impedance (i.e., high impedance indicates gas or air, low impedance indicates liquid). The signals from six impedance channels and one pH channel were recorded at 50 samples per second. The data were stored in an ambulatory recorder and saved on a 256 MB Compact Flash card. The study was performed as an outpatient design after an overnight fast with LES that was located by esophageal manometry. All patients were at least 7 days off PPI therapy and H2 receptor antagonist treatment, and 3 days off β receptor agonist, aminophylline and nitrate therapy.

Acid reflux events were defined as the point at which the distal pH dropped to below pH 4 (acid). Non-acid reflux events were detected as retrograde reductions in the impedance signal representing liquid reflux events with a pH > 4. Acid exposure time (%) was defined as the total time at pH < 4 divided by the time monitored. Liquid bolus entry was the time at which a 50% fall in impedance from the baseline defining the liquid reflux was reached. Bolus duration was the time between liquid bolus entries to the time of liquid bolus clearance, which was defined as the moment when impedance increased to values denoting liquid reflux entry for ≥ 5 s. Bolus duration was measured at the impedance site at a height of 5 cm above the LES. Bolus exposure time (%) was defined as the bolus duration time divided by the time monitored [10]. Distal reflux events were measured by the impedance site that was located 5 cm above the LES, and proximal reflux events were measured by the impedance site that was located 15 cm above the LES.

### Comparison groups

IPF Patients were divided into GERD+ and GERD- groups according to the results of the 24-hour pH monitoring process [10]. Abnormal upright (≥6.3%) or recumbent (≥1.2%) or total (≥4.2%) acid exposure time and/or a Demeester score greater than 14.7 [14]. Values of HRiM between those found for IPF patients and those found for healthy volunteers were compared, and values of HRiM and MII/pH between GERD patients and those without IPF were also compared.

### Statistical methods

Categorical data were described as singular data points, and continuous data as mean ± SD. Data were analyzed by one-sample Student’s t test, independent sampled t test or Chi-square test. A p value <0.05 was considered as statistically significant. All data were analyzed with SPSS 17.0 software analysis tool.

## Results

Between July 2011 and July 2013, 346 patients undertook HRiM and MII/pH therapy, and 88 of the patients were treated with IPF. In addition, 19 of them also presented with coronary artery disease, diabetes and connective tissue diseases, and 69 patients that presented with IPF were enrolled to the study. Between January 2010 and January 2011, 62 healthy volunteers were enrolled to the study in our department to measure the normal values of HRiM in the population of northern China, and these patients were enrolled as a control group of HRiM treated subjects. After analyzing the results of all 346 patients, 88 patients without coronary artery disease, diabetes, lung diseases and connective tissue diseases, were diagnosed with GERD and were then subsequently enrolled as a positive control group of GERD patients.

The demographic data and symptoms of patients with IPF are shown in Table 1. Also, 43 patients were in the GERD+ group and 26 patients were in GERD- group. There were no significant differences in terms of age, gender, former smoking behavior, duration of disease, FVC, or DLCO (*P* > 0.05). Both groups of patients also presented with similar symptoms.

**Table 1:** **Demographic data and symptoms of different groups**

Items	GERD+*n* = 43	GERD-*n* = 26	Sensitivity Specificity %	PPV NPV %	Independent-Sample t test or Chi-square
Age (mean ± SD, yr)	57.9 ± 10.3	62.5 ± 10.1	-	-	*P* = 0.077
Male/ Female, n (% )	19/24	12/14	-	-	*P* = 0.873
Former smoker , n	21	10	-	-	*P* = 0.431
FVC (%, mean ± SD)	65.3 ± 6.4	64.2 ± 7.1	-	-	*P* = 0.557
DlCO (%, mean ± SD)	51.6 ± 9.1	48.8 ± 7.5	-	-	*P* = 0.194
Duration of disease (mean ± SD, month)	22.8 ± 23.2	16.4 ± 18.3	-	-	*P* = 0.231
Heartburn, n (%)	25(58.1)	9(34.6)	58.1	73.5	*P* = 0.058
			65.4	48.6	
Chest Pain, n (%)	3(6.9)	1(3.8)	6.9	75.0	*P* = 0.168
			96.2	38.5	
Regurgitation, n (%)	1(2.3)	1(3.8)	2.3	50.0	*P* = 0.715
			96.2	37.3	
Any typical symptom	25(58.1)	10(38.4)	58.1	71.4	*P* = 0.113
			61.6	47.1	
Cough, n (%)	40(93.0)	25(96.1)	93.0	61.5	*P* = 0.590
			3.9	25.0	
Dyspnea on exertion, n (%)	38(88.0)	22(84.6)	88.4	63.3	*P* = 0.653
			15.4	44.4	
Belch, n (%)	6(13.9)	1(3.8)	13.9	85.7	*P* = 0.178
			96.2	40.3	
Difficulty swallowing, n (%)	1(2.3)	0(0)	2.3	100	*P* = 0.433
			100	38.2	
Epigastric pain, n (%)	2(4.6)	0(0)	4.6	100	*P* = 0.264
			100	38.8	

GERD: gastro-esophageal reflux disease; FVC: forced vital capacity; DLCO: diffusing capacity of the lung for carbon monoxide; PPV: positive predictive value; NPV: negative predictive value.

The results of HRiM between patients with IPF and healthy volunteers are shown in Table 2. Compared with healthy volunteers, patients with IPF showed a pattern of a significantly older age, significantly lower LESP, UESP, DCI, DEA, and CBTR, while they also showed significantly higher TBTT and prevalence of weak peristalsis.

**Table 2:** **Results of high-resolution manometry and impedance**

Metrics	IPF patients	Healthy volunteers	Independent-Sample t test or Chi-square test
	*n* = 69	*n* = 62	
	Mean ± SD	Mean ± SD	
Age	57.9 ± 10.3	32.0 ± 11.3	*P* < 0.001
Gender (M/F)	31/38	25/37	*P* = 0.595
LESP mm Hg	17.4 ± 8.8	26.3 ± 10.8	*P* < 0.001
Hypotensive LES n (%)	13	2	*P* = 0.005
LESRP mm Hg	4.0 ± 5.0	6.6 ± 4.7	*P* = 0.008
IRP mm Hg	8.1 ± 4.6	10.4 ± 4.9	*P* = 0.008
UESP mm Hg	77.7 ± 39.2	101.4 ± 49.5	*P* < 0.001
DEA mm Hg	59.3 ± 37.0	95.3 ± 35.4	*P* < 0.001
DCI mm Hg.cm.s	754.1 ± 720.9	1891 ± 1131	*P* < 0.001
Hiatal hernia n (%)	7 (10.1)	1 (1.6)	*P* = 0.046
Peristalsis n (%)			
Normal	37 (53.6)	57 (91.9)	*P* < 0.001
Weak	32 (46.4)	5 (8.1)	
TBTT s	7.5 ± 1.4	6.9 ± 0.9	*P* = 0.016
CBTR %	78.0 ± 20.5	90.3 ± 14.0	*P* < 0.001

Key:

IPF: idiopathic pulmonary fibrosis; LESP: lower esophageal sphincter pressure; LES: lower esophageal sphincter; LESRP: lower esophageal sphincter residual pressure; IRP: integrated relaxation pressure. UESP: upper esophageal sphincter pressure; DCI: distal contractile integral; DEA: distal esophageal amplitude; TBTT: total bolus transmit time; CBTR: complete bolus transit rate.

Abnormal proximal reflux events were observed in 37.7% (26/69) patients with IPF, and abnormal distal reflux events were observed in 33.3% (23/69) of patients (Table 3). However, a common abnormality was non-acid reflux, and thus 71% of patients had a negative DeMeester score. Comparisons of GERD patients with and without IPF are shown in Table 4. GERD patients with IPF showed significantly lower UESP, significantly lower CBTR, and significantly higher bolus exposure times. By contrast, GERD patients with IPF showed no statistical difference of LESP, weak peristalsis, acid exposure time, distal and proximal reflux events.

**Table 3:** **24-hour multi-channel intraluminal impedance and pH measurements**

Measurements n = 69	N (%)
Abnormal acid exposure time	43 (62.3)
Upright ≥6.2%	18 (26.0)
Recumbent ≥1.2%	28 (40.6)
Total ≥4.2%	20 (29.0)
Total proximal reflux events: acid	714 (50.7)
Non-acid	695 (49.3)
Patients with abnormal proximal reflux events	26 (37.7)
Acid reflux event ≥28	5 (7.2)
Non-acid reflux event ≥13	22 (31.9)
Total reflux event ≥31	15 (21.7)
Total distal reflux: acid	1187 (43.8)
Non-acid	1524 (56.2)
Patients with abnormal distal reflux events	23 (33.3)
Acid reflux event ≥55	0 (0)
Non-acid reflux event ≥27	23 (33.3)
Total reflux event ≥73	5 (7.2)
DeMeester score: positive	20 (29.0)
Negative	49 (71.0)

Twenty-four hour ambulatory simultaneous impedance and pH monitoring: a multi-center report of normal values from 60 healthy volunteers [10].

**Table 4:** **Comparisons of GERD patients with versus without IPF (mean ± SD)**

Items		GERD with IPF	GERD without IPF	Independent-Sample t test
		*N* = 43	*N* = 88	
Age		57.9 ± 10.2	54.2 ± 12.3	*P* = 0.097
Gender		19/24	39/49	*P* = 0.989
LESP (mean ± SD)		16.7 ± 9.4	16.3 ± 11.7	*P* = 0.811
LESRP (mean ± SD)		4.0 ± 5.0	4.8 ± 4.5	*P* = 0.372
IRP (mean ± SD)		8.2 ± 5.1	7.9 ± 5.6	*P* = 0.717
UESP (mean ± SD)		66.3 ± 16.5	78.0 ± 29.6	*P* = 0.018
DEA (mean ± SD)		59.3 ± 37.0	61.6 ± 31.3	*P* = 0.713
DCI (mean ± SD)		792.2 ± 838.3	592.4 ± 746.4	*P* = 0.169
Peristalsis n (%)				
Normal		24 (55.8)	53 (60.2)	*P* = 0.630
Weak		19 (44.1)	35 (39.8)	
TBTT s		7.4 ± 1.6	7.0 ± 1.3	*P* = 0.115
CBTR %		56.0 ± 37.0	69.4 ± 34.0	*P* < 0.001
Acid exposure (pH)		Percent time (%)		
Upright		6.4 ± 8.2	5.4 ± 5.7	*P* = 0.405
Recumbent		4.3 ± 5.9	2.6 ± 4.2	*P* = 0.099
Total		4.8 ± 4.2	4.2 ± 3.8	*P* = 0.403
DeMeester score		15.9 ± 12.6	15.0 ± 12.8	*P* = 0.713
Bolus exposure		All reflux time (%)		
Upright		4.6 ± 3.4	3.0 ± 2.5	*P* = 0.008
Recumbent		1.5 ± 2.1	0.6 ± 1.3	*P* = 0.022
Total		2.8 ± 2.0	1.9 ± 1.7	*P* < 0.009
		Reflux Episodes Activity		
Distal	Acid	21.1 ± 11.3	21.3 ± 14.8	*P* = 0.927
Reflux	Nonacid	24.3 ± 14.8	20.1 ± 14.5	*P* = 0.199
n	Total	45.5 ± 17.9	41.4 ± 23.7	*P* = 0.326
Proximal	Acid	13.0 ± 9.2	12.1 ± 10.1	*P* = 0.523
Reflux	Nonacid	11.0 ± 8.5	11.1 ± 10.6	*P* = 0.615
n	Total	24.3 ± 14.8	23.1 ± 17.3	*P* = 0.951

GERD: gastro-esophageal reflux disease; IPF: idiopathic pulmonary fibrosis; LESP: lower esophageal sphincter pressure; LESRP: lower esophageal sphincter residual pressure; IRP: integrated relaxation pressure. UESP: upper esophageal sphincter pressure; DCI: distal contractile integral; DEA: distal esophageal amplitude; TBTT: total bolus transmit time; CBTR: complete bolus transit rate.

## Discussion

The last ten years has seen considerable advances in the sensitivity of techniques to study esophageal motor disorders and GERD, which have collectively revealed clinical features that can help explore the pathophysiology of various conditions in great detail [9,10]. Impedance monitoring is a new method that allows detection of bolus transit. It does so by detecting a change in the resistance to current flow between pairs of electrodes when a liquid and/or gas bolus bridges them. When a liquid bolus bridges the two electrode rings, impedance decreases directly with the degree of bolus ionization. By contrast, a gas bolus increases impedance. These differences in impedance characteristic allows the differentiation of liquid, gas and combined liquid–gas (mixed) boluses. In addition, combining impedance with pH monitoring can determine bolus exposure time, composition, and proximal extent of the refluxate. Both of these measures can be used to assess the risk for micro-aspiration [7].

In this study, we found that the prevalence of GERD in Chinese patients with IPF was high (i.e., 62.3%), but GERD symptoms were found to be a poor predictor of the presence of reflux within the group. The sensitivity and specificity of any typical symptoms (e.g., heartburn, regurgitation and chest pain) was 58.1% and 61.6%, positive predictive value, and the negative predictive value was 71.4% and 47.1%. These data confirm that Chinese patients with IPF have a similar profile as those seen in other countries in previous studies [4,15-17]. The relatively poor sensitivity and specificity of using symptoms alone outlines the importance of performing objective assessments of esophageal function and reflux status in IPF as opposed to empirically treating GERD in the entire cohort.

Although the cause of IPF remains unknown, several associations have been described, including cigarette smoking, exposure to wood and metal dusts, chronic viral infection, exposure to some drugs (e.g., antidepressants) and hereditary factors and mutations in the genes encoding telomerase components [2,18]. Recent emerging data supports a role for chronic micro-aspiration (e.g., subclinical aspiration of small droplets due to GERD) which may cause repetitive subclinical injury to the lung leading to pulmonary fibrosis [3,7]. Studies [19,20] have been described that show IPF patients that presented with increased prevalence of hiatal hernia and IPF patients that presented with hiatal hernia had a greater physiological impairment on pulmonary function testing. Mays et al. [21] suggested that repeated small tracheobronchial aspiration of gastric secretions over a long period of time could lead to lung fibrosis. Lee et al. [22] detected pepsin, a biomarker of micro-aspiration, in bronchial alveolar lavage (BAL) fluid from patients with IPF, and it was direct evidence that showed that gastric contents can reach the lower respiratory tract without an overt aspiration. The observed association of hiatal hernia and abnormal GERD coupled with the detection of pepsin in BAL specimens retrieved from the distal pulmonary parenchyma supports the long-standing hypothesis that micro-aspiration is a causative role in the development of IPF and may trigger episodes of acute execrations of IPF.

For several decades, esophageal specialists have postulated that altered respiratory mechanics in patients with end-stage lung disease may cause reflux. Specifically, increases in positive intra-abdominal pressure and negative intrathoracic pressure (with a corresponding increase in the transdiaphragmatic pressure gradient) could cause a gradient-favoring reflux [23,24]. Survival analysis [25] showed that identifying the presence of gastro-esophageal symptoms, providing a reflux diagnosis, medication use, and Nissen fundoplication were all collectively associated with longer survival in IPF.

In this study, we also found that patients with IPF presented with lower LESP and UESP, higher prevalence of hypotensive LESP and hiatal hernia, and combined impedance showed that in addition to 46.4% of patients having weak peristalsis, 33.3% of patients had abnormal distal reflux events, 37.7% of patients had abnormal proximal reflux events, and moreover, IPF patients presented with higher TBTT and lower CBTR which meant that the bolus transit was impaired.

The esophagus comprises striated muscle in the proximal third of the esophageal body and smooth muscle in the distal two-thirds. Where these two types of muscle meet there is a ‘transition zone’ which can lead to a ‘break’ in the peristaltic wave as it travels distally. This occurs in healthy individuals under normal conditions, but it has been shown that if this break is greater than 2 cm, it can lead to the bolus not being completely transported and cleared into the stomach. In patients with weak peristalsis, these breaks can often be characterized as small (2–5 cm) or large (>5 cm). It has been demonstrated that among healthy volunteers weak peristalsis of the esophageal body has predicted incomplete bolus clearance [26] and might account for delayed bolus transit and impaired esophageal reflux clearance in patients with GERD [27,28]. In addition, weak peristalsis with large breaks is associated with high acid exposure and delayed reflux clearance in the supine position in GERD patients [28]. Moreover, GERD patients with IPF, have presented with lower UESP, lower CBTR and a higher bolus exposure time than GERD patients without IPF. This means that there is an increasing possibility of small droplets from the esophagus that are capable of being aspirated into the trachea, and supports the hypothesis that GERD and chronic micro-aspiration may play a major role in the pathobiology of IPF.

GERD has long been known to be an important cause of chronic cough. Indeed a diagnosis of gastro-esophageal acid reflux-associated cough (GERC) is often made following a successful, empirical trial of high-dose acid suppression therapy. Thus, a particular attraction of the reflux hypothesis is that it could explain both the pathogenesis of IPF and the associated symptom of cough. However, the lack of improved cough symptoms despite effective acid suppression therapy indicates that gastroesophageal acid reflux alone is not wholly responsible for cough in the majority of patients with IPF. A recent advance in the assessment of non-acid reflux is the technique of combined esophageal pH and impedance monitoring, which provides a means of detecting acid and non-acid reflux. Savarino et al. [29] reported a high frequency of occurrence of acid reflux events, non-acid reflux events, and reflux reaching the proximal esophagus in patients with systemic sclerosis and interstitial lung disease. Our study also found that patients with IPF had more distal and proximal reflux events, especially non-acid reflux, which showed combined impedance and pH recordings that provided a more accurate assessment of the specific pattern of reflux.

Mise et al. [30] reported lower DLCO in patients with recently diagnosed GERD in comparison with healthy controls. Bonacin et al. [31] showed statistically significant increases in FVC in the GERD group as compared with the non-GERD group. Among the GERD group, values of DLCO and DLCO/VA were significantly lower, and intrapulmonary shunt was significantly higher in comparison with the non-GERD group, which confirmed the correlation between GERD and damaged lung function. The results of that study suggested an additional pathological mechanism in the development of intrapulmonary shunt, due in part, to microatelectasis resulting from surfactant damage caused by micro-aspiration of the stomach contents. Both of the aforementioned studies stated the need for early lung function tests in all GERD patients. In our study, esophageal function parameters in IPF patients presenting with GERD did not correlate with worsened pulmonary function (FVC or DLCO). We also did not find a significant correlation in our research between FVC or DLCO and motility or reflux. This may be due to the relatively small numbers of research subjects that were recruited to this study (i.e., when comparing quite variable parameters, as the study was not powered to detect these changes), and the inclusion of IPF patients at the milder end of the spectrum. Some researchers [32-34] have showed that anti-reflux surgery can improve lung function of GERD patients so further studies are required to determine whether anti-reflux surgery or using PPI might improve the prognosis of IPF patients.

Our study also has some limitations. All subjects were recruited from one hospital and in only one city, which might have a potential selection bias. Besides, the small number of patients limited the statistical power of the study. The life signs of all 346 patients were stable but in some of the severe presenting patients who needed inspired oxygen management, we decided that they should not undertake the procedure. Whilst this means there is somewhat the risk of a selection bias for patients at the mild end of the IPF spectrum, we felt justified to use these inclusion criteria on the grounds of patient safety and to exclude the confounders of other underlying diseases (e.g., connective tissue disease) to esophageal motility. Healthy Chinese volunteers were younger than IPF patients, and we used the diagnostic criteria of abnormal acid exposure from the USA. However, we do have sufficient merit to demonstrate that it is important to screen GERD in IPF patients, even if there do not present with typical reflux symptoms.

## Conclusions

GERD prevalence in Chinese patients with IPF was high, whereas GERD symptoms were a poor predictor of GERD being present. IPF patients had lower LESP and UESP, impaired esophageal peristalsis and bolus clearance function, and a higher frequency of proximal reflux events. Combined MII/pH should be considered the ‘gold standard’ in assessing reflux in IPF.
